# Risk of complications in late term pregnancies adjusted for induction of labor

**DOI:** 10.1111/aogs.14357

**Published:** 2022-04-05

**Authors:** Charlotte Brix Andersson, Jesper Padkær Petersen, Søren Paaske Johnsen, Ulrik Schiøler Kesmodel

**Affiliations:** ^1^ Danish Center for Clinical Health Services Research (DACS) Aalborg Denmark; ^2^ Department of Obstetrics and Gynecology Aalborg University Hospital/Thisted Denmark; ^3^ Department of Pediatrics Aarhus University Hospital Aarhus Denmark; ^4^ Department of Obstetrics and Gynecology Aalborg University Hospital Aalborg Denmark

*Sir*,

We thank Drs Rasmussen and Lauszus for their comments^1^ on our article.^2^ We believe they may have misunderstood the aim of this study. The study was not “concerning induction of labor (IOL) in post term pregnancies”. In fact, the aim of our study was to investigate whether gestational age (GA) per se increases the risk of complications for the child and the mother in late term pregnancies independent of IOL and other factors.

You are quite right about the influence of IOL on the crude risk of adverse outcomes. The same is the case for the other factors that influence the risk of complications,such as maternal body mass index, maternal age and parity. By adjusting for all these factors, including IOL, in the regression analyses, we aimed to reduce relevant confounding as much as possible, and we believe that this is the most accurate way to investigate the influence of GA on the risk of complications within the GA span of interest. Please see the causal model of the study.

Whether IOL increases the risk of cesarean section (CS) (or other adverse obstetric or neonatal outcomes) is also an interesting question, but it was not within the scope of our study. Even so, if the risk of adverse outcomes is systematically increased with increasing GA, as is the case, this would speak in favor of somehow advocating for IOL to the extent that adverse effects of IOL do not exceed those of increasing GA.

We would be cautious when discussing a potentially increased risk of CS after IOL. You refer to a study from 2003 based on a historical cohort delivered in Boston during 1998 and 1999.^3^ In the subsequent observational study by Caughey et al^3^ that you cite, no increased risk of CS was observed after IOL after weeks 39–40. Also, more recent randomized trials showed no difference or a decreased risk of CS when comparing IOL and expectant management in late term pregnancies.^4^, ^5^ Because of the huge risk of confounding, it is difficult to compare induced and not induced births in an observational study design.
